# Supplementary material for: Stacking Model‐Based Classifiers for Dealing With Multiple Sets of Noisy Labels
Source: Biom J. 2025 Mar 12;67(2):e70042. doi: 10.1002/bimj.70042 (PMC11898607; doi:10.1002/bimj.70042)
Supplement: Supplementary file 1 — Supporting Information [file BIMJ-67-e70042-s001.zip › density-based-ensemble-model-main/competitors_real_data/netlab-master/netlab-master/help/mdn2gmm.htm]

Netlab Reference Manual mdn2gmm


# mdn2gmm

## Purpose

Converts an MDN mixture data structure to array of GMMs.

## Synopsis

```
gmmmixes = mdn2gmm(mdnmixes)
```

## Description

`gmmmixes = mdn2gmm(mdnmixes)` takes an MDN mixture data structure
`mdnmixes`
containing three matrices (for priors, centres and variances) where each
row represents the corresponding parameter values for a different mixture model
and creates an array of GMMs. These can then be used with the standard
Netlab Gaussian mixture model functions.

## Example

```
mdnmixes = mdnfwd(net, x);
mixes = mdn2gmm(mdnmixes);
p = gmmprob(mixes(1), y);
```

This creates an array GMM mixture models (one for each data point in
`x`). The vector `p` is then filled with the conditional
probabilities of the values `y` given `x(1,:)`.

## See Also

`gmm`, `mdn`, `mdnfwd`

---

**Pages:**
Index


---

Copyright (c) Ian T Nabney (1996-9)

David J Evans (1998)
